# A Rare Case of Idiopathic Pleuro-Parenchymal Fibroelastosis Presenting With Bilateral Spontaneous Pneumothoraces

**DOI:** 10.7759/cureus.56975

**Published:** 2024-03-26

**Authors:** Prasad Kumar, Shahnawaz Hashmi, Matthew Birbeck, Mohamed Seklani, Nilaa Subramanian

**Affiliations:** 1 Pulmonology, University Hospitals Bristol and Weston NHS Foundation Trust, Bristol, GBR; 2 Internal Medicine, University Hospitals Bristol and Weston NHS Foundation Trust, Bristol, GBR; 3 Pulmonary Medicine, University Hospitals Bristol and Weston NHS Foundation Trust, Bristol, GBR

**Keywords:** pneumothorax (ptx), rapidly progressive, interesting case report, spontaneous bilateral pneumothorax, fibroelastosis, pleuroparenchymal

## Abstract

Pleuroparenchymal fibroelastosis (PPFE) is a rare interstitial lung disease (ILD), characterized by predominantly upper lobe pleural and subjacent sub-pleural parenchymal fibrosis. Its name refers to a combination of fibrosis involving the visceral pleura with fibroelastotic changes, predominantly in the subpleural lung parenchyma.

We describe the case of a 67-year-old lady who presented to the accident and emergency department of Weston General Hospital with worsening shortness of breath (SOB) and cachexia of six to eight months' duration.

The initial imaging studies showed bilateral spontaneous pneumothoraces on a background of pleural-based consolidation and fibrotic changes. A subsequent high-resolution CT (HRCT) chest showed evidence of pleuroparenchymal fibroelastosis in the background. She was not considered for anti-fibrotic medications due to the advanced stage of the disease and was managed with supportive measures, including oxygen support, oral steroids and antibiotics to cover for any infections. After initial management of symptoms and long discussions with the patient, family and the palliative team, she was discharged home with community follow-up.

## Introduction

Pleuroparenchymal fibroelastosis (PPFE) is an uncommon form of pulmonary fibrosis marked by fibrosis primarily in the upper lobes, exhibiting distinctive histopathological features such as thickening of the visceral pleura with collagenous fibrosis, elastosis beneath the pleura, and collagenous fibrosis within the alveoli.

Typical symptoms upon presentation include difficulty breathing, a persistent dry cough, weight loss, and chest discomfort. Patients with PPFE often display a slender physique and a flattened chest [1]. 

Since 2013, it has been recognized as a rare subtype of interstitial lung disease, acknowledged in the joint diagnostic guidelines for interstitial lung diseases published by the American Thoracic Society (ATS) and the European Respiratory Society (ERS) [2].

Presently, there are two recognized types of pleuroparenchymal fibroelastosis: the idiopathic variant, where a specific cause cannot be determined, and a secondary type linked to various underlying factors.

Distinguishing features such as an early-onset age, low body mass index, flat chest configuration, prevalence in the upper lobes, increased occurrence of pneumothorax, and bronchopleural fistulae help differentiate this condition from idiopathic pulmonary fibrosis [3-4].

Pleuroparenchymal fibroelastosis may be seen in 6-10% of cases of Idiopathic pulmonary fibrosis (IPF) [4-5]; it may be associated with a more rapid decline in lung function, higher risk of pneumothorax and pneumomediastinum, and poorer survival [5].

In this article, we describe a case of a 67-year-old female who presented to our accident and emergency department with a six- to eight-month history of dyspnoea and cachexia. Imaging performed on admission revealed pneumothoraces bilaterally and a subsequent HRCT showed findings characteristic of PPFE. As per our literature review on PubMed, Google Scholar and other platforms, this is the first documented case of PPFE presenting with bilateral spontaneous pneumothorax in the UK.

## Case presentation

A 67-year-old female presented to the accident and emergency department of our hospital in February of 2023 with worsening shortness of breath (SOB), nonproductive cough and left upper quadrant pain. The patient reported that the SOB and cough were present since October 2022 and her general practitioner (GP) had started her on a weaning course of prednisolone, based on chest X-ray (CXR) findings suggestive of interstitial lung disease (ILD). On admission, the said dose was 5mg once daily (OD). Her exercise tolerance (ET) was very limited on admission. She also reported a significant weight loss of 19 kg in this time period. The patient had never had any respiratory symptoms in the past apart from the current illness.

As per social and occupational history, she used to work in a shoe factory and was never exposed to asbestos. She lived with her husband and was fully independent before the current illness. She kept cockatiels as pets for 18 years; however, she never had any related symptoms. There was no history of any allergies.

On initial assessment on admission, she appeared very frail with a low body weight of 41 kg (she was 60 kg one year prior), flat chest wall with prominent rib spaces, and reduced chest expansion with breathing. There was tachypnoea with a respiratory rate (RR) of 36/min, saturations were 94% on room air, heart rate (HR) of 91/min, blood pressure of 127/82 mmHg, and normal temperature. Chest auscultation revealed diffuse bilateral fine crackles. Abdominal examination was unremarkable. Arterial blood gas (ABG) on admission showed normal pH: 7.43 with normal pCO2 of 6.12 kPa, PaO2 of 10.9 kPa and saturation (SO2) of 97%. Blood work and other investigations were done, which are shown in Table 1.

**Table 1:** Initial blood investigations Hb: hemoglobin; WCC: white cell count; CRP: C-reactive protein; CEA: carcinoembryonic antigen; AFP: alpha fetoprotein; ANCA: antineutrophil cytoplasmic antibodies; RAST: radioallergosorbent test; Anti-DsDNA: anti-double stranded DNA; ACE: angiotensin converting enzyme

Parameters	Patient results	Reference range
Hb	148 g/L	120-150 g/L
WCC	12.32 x10^9 ^/ L	4.0-11.0 x 10^9^/L
Platelets	420 x10^9 ^/L	150-400 x 10^9^ /L
CRP	<5 mg/dL	<6mg/dL
D-dimers	590 ng/ml	<=680 ng/ml
Calcium	2.73 mmol/L	2.20-2.60 mmol/L
HIV	Negative	
Serum electrophoresis	No paraprotein detected	
Rheumatoid factor	Negative	0-20 IU/mL
CEA	Negative	0- 2.9 ng/mL
AFP	Negative	5- 10 ng/mL
Ca 19-9	Negative	<35 units/mL
Ca-125	91 kU/L	<35 units/mL
Ca 15-3	100 kU/L	<25 units/mL
ANCA	Negative	
RAST profile for allergens	All Negative	
Total IgE	33 kU/L (normal)	1-113 kU/L
Anti-DsDNA	<9.8 Negative	<27 negative
Serum ACE	<3 U/L	8-25 U/L
Aspergillus precipitants	Grade 0 - Negative	Grade 0 - negative; Grade 1-2 weak positive; Grade 3-4 moderate positive; Grade 5-6 strong positive

**Figure 1:**
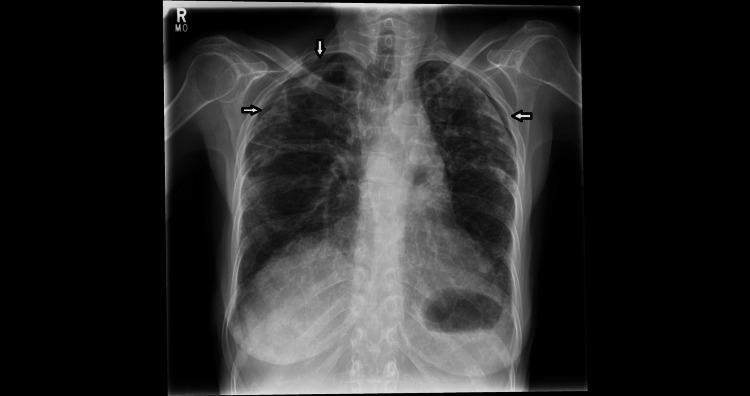
There is bi-apical pleural thickening, pleuro-parenchymal thickening, upper lobe volume loss and superior retraction of the hila. Note the small bilateral apical pneumothoraces in the CXR on admission (white arrows). CXR: chest X-ray

CXR was done (Figure 1), revealing bilateral apical pneumothoraces (less than 2 cm) and underlying changes of fibrotic lung disease. 

**Figure 2:**
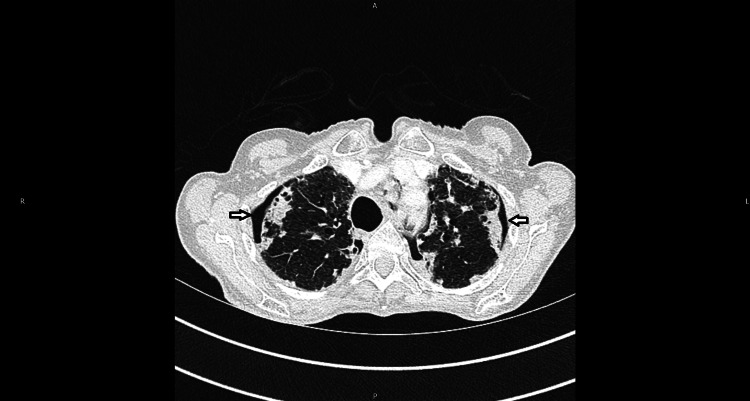
CT thorax demonstrating biapical pleural thickening, subpleural fibrosis and traction bronchiectasis. Note the bilateral pneumothoraces (arrow).

CT-thorax, abdomen and pelvis (TAP) were performed to look for any evidence of malignancy, given the significant weight loss and cachexia. CT-TAP (Figure 2) showed bilateral apical pneumothoraces with underlying biapical pleural thickening and subpleural fibrosis with traction bronchiectasis. There was a right ovarian mass of 3.5 cm, suggestive of fibroma.

A referral to the thoracic surgical team was made to seek advice on the management of bilateral pneumothoraces. The advice was to manage conservatively as the patient was not fit for any invasive surgical procedures. The ovarian mass was later discussed in the gynaecology multi-disciplinary team (MDT) meeting, which concluded that the lesion was in keeping with an ovarian fibroma. The MDT outcome was for conservative management with surveillance, given the poor performance status due to the extensive lung disease.

The CT chest images were discussed in the hospitals’ ILD MDT and the scans were in keeping with PPFE. Bronchoscopy and bronchoalveolar lavage (BAL) were not done as the patient was at risk of complications due to bilateral pneumothorax. Also, she was not considered for antifibrotics due to the advanced stage of the disease. The recommendation was for symptom control measures, such as oxygen support, oral morphine for anxiety and SOB, nutritional support, and palliative care. She was also deemed not suitable for a lung transplant, considering her advanced age (>60), BMI less than 17, and the underlying ovarian mass (high CA-125). The role of prophylactic antibiotics to prevent recurrent infections was also considered. The role of azithromycin prophylaxis was considered in view of the possibility of infection due to being on weaning steroids since October 2022. However, this was not started as there was no evidence of infection in her blood work. The CRP was within normal limits and she did not show signs of sepsis.

A six-minute walk test was done which showed that her maximum Borg score was 2. The distance covered during the test was 240 meters; oxygen saturation was 96% on room air which dropped to 90% at the end of the test. After optimizing the supportive measures, with patient and family counselling, she was discharged home on the seventh day of admission. She was discharged on a weaning dose of prednisolone, carbocisteine, oral morphine, proton pump inhibitors (PPIs) and dietary supplements. On discharge, she was dependent on her husband for most of her daily activities. Notably, she was completely independent prior to the current illness.

Following her discharge, she had two admissions in the subsequent two months, each time presenting with worsening SOB and chest pain. Her serial ABG measurements in these admissions showed rising PCO2; however, the pH remained normal. Her BMI continued to decrease and on the final admission, her BMI was 15.7. She was managed with supportive measures every time, as per the previous plan. Sadly, she passed away peacefully at her home four months after her initial discharge (following the last admission in April), due to her progressive disease and overall poor condition.

## Discussion

PPFE was first described in Japan by Amitani et al. [6] as idiopathic pulmonary upper lobe fibrosis (PULF). It was called Amitani disease until the term PPFE was coined by Frankel et al. in 2004 [1].

The primary diagnostic distinctions for PPFE encompass disorders linked with upper lobe involvement, such as hypersensitivity pneumonitis, sarcoidosis, idiopathic interstitial pneumonia extending into upper zones (including UIP), atypical conditions like nontuberculous mycobacterial infection, post-lung injury remodelling, pneumoconiosis, malignancy, and apical pleural cap. The most frequent fibrotic ILD pattern found concurrently with PPFE is usual interstitial pneumonia (UIP), observed in approximately a quarter to half of cases [4]. Coexistent UIP or even nonspecific interstitial pneumonia (NSIP) occurs most frequently in the lower lobes, away from the main areas of PPFE, but in common with the latter, each pattern will typically progress over time [7-8]. PPFE has also been reported in patients diagnosed with chronic hypersensitivity pneumonitis (HP) [9-10]. 

Anteroposterior flattening of the chest, or platythorax, occurs commonly in PPFE [1] and has been correlated with decreased BMI. This has been observed in our case, as described above. Although PPFE has been reported in children and the elderly, most patients are between 40 and 70 years of age. Younger patients were overall more likely to be female. This was observed in our patient who was a 67-year-old female.

The duration of symptoms before presentation varies from 6-24 months. The most common of these are progressive breathlessness and cough; nonspecific chest discomfort and pleuritic pain are reported, but persisting pain is unusual in the absence of pneumothorax. Progressive weight loss is frequently reported during the disease course and may raise the possibility of an intercurrent infection or occult malignancy. In the case we described, symptoms presented over a six-to-eight-month period and were associated with significant weight loss of 19 kgs over a year. There was also an underlying ovarian mass which was concluded to be an ovarian fibroma. The progression of PPFE can vary significantly, with a median survival time of around 11 years. However, in certain individuals, the disease can advance rapidly, resulting in a mean survival of three to five years [11]. The patient in our report above had a rapidly progressive disease and died within six months of presentation.

No treatment has been shown to be effective in PPFE. Low-dose prednisolone, used empirically, may produce useful, although unproven, immunomodulatory effects [12]. Administration of higher doses of corticosteroids or immunosuppressive drugs like azathioprine or methotrexate is typically avoided due to the elevated susceptibility to infections in these individuals. Previously perceived as a slowly advancing condition, it's now recognized that certain PPFE patients experience an inevitably progressive trajectory, leading to irreversible respiratory failure and premature death. Without effective pharmaceutical interventions, lung transplantation stands as the sole therapeutic recourse for this ailment. A portion of patients grappling with progressive pulmonary fibrosis may find relief through antifibrotic medications, provided they meet the eligibility criteria [13]. The patient in discussion was initially tried on low-dose weaning steroids, which were not of significant benefit. She was not considered for antifibrotics due to the advanced stage of her disease, and a lung transplant was not an option due to the relative contraindications (BMI <17, age >60, with underlying ovarian mass).

The patient described in this case presented to the hospital for the first time with bilateral spontaneous pneumothoraces, which is an uncommon presentation. The only previous case reported with a similar presentation was a 46-year-old lady with bilateral pneumothoraces by Maturu et al. [14] from India. Another case report, by Zhang et al. [15] from China, described a patient with a unilateral pneumothorax, and recurrent bilateral pleural effusion. Kinoshita et al. from Japan described a 78-year-old man presenting with bilateral pneumothorax who was found to have underlying PPFE [16]. In general, bilateral pneumothoraces are extremely unusual and represent only 1.5% of all pneumothorax cases [17]. Following an extensive literature search, we can say that, to the best of our knowledge, our patient is the first case reported in the UK with bilateral pneumothoraces in PPFE on the first presentation.

In PPFE patients, pneumothorax is a frequent occurrence, with a three-year cumulative rate reaching 53.9%. Remarkably, 15.8% of PPFE instances exhibited bilateral pneumothoraces, reflecting the refractory nature of the condition, often resulting in contralateral pneumothorax prior to healing [18]. Despite this, initial bilateral spontaneous pneumothorax incidents are infrequent in reported cases. In PPFE, pneumothorax arises from the progressive upper lung lobe contraction and is typically left untreated due to the confined space and resistance to thoracic drainage [18]. 

## Conclusions

In conclusion, to our knowledge, we are the first to report a case of bilateral pneumothoraces as an initial presentation in a 67-year-old with PPFE. PPFE is a rare form of ILD with limited treatment options. Additional work is needed to better understand this rare condition in order to come up with potential therapeutic agents to manage this condition conservatively.

Furthermore, future work is warranted to establish the incidence and treatment options of pneumothoraces in individuals with PPFE. Lastly, PPFE should also be considered as a differential diagnosis in patients (with similar demographics) presenting with bilateral pneumothoraces.
